# Development and validation of a prognostic model based on comorbidities to predict COVID-19 severity: a population-based study

**DOI:** 10.1093/ije/dyaa209

**Published:** 2020-12-08

**Authors:** Francisco Gude-Sampedro, Carmen Fernández-Merino, Lucía Ferreiro, Óscar Lado-Baleato, Jenifer Espasandín-Domínguez, Xurxo Hervada, Carmen M Cadarso, Luis Valdés

**Affiliations:** 1 Departamento de Epidemiología. Complejo Hospitalario Universitario de Santiago de Compostela. Santiago de Compostela, Spain; 2 Grupo de Métodos de Investigación, Instituto de Investigaciones Sanitarias de Santiago (IDIS), Santiago de Compostela, Spain; 3 Departamento de Medicina Familiar y Comunitaria. Centro de Saúde A Estrada. Pontevedra, Spain; 4 Servicio de Neumología. Complejo Hospitalario Universitario de Santiago de Compostela. Santiago de Compostela, Spain; 5 Grupo Interdisciplinar de Investigación en Neumología. Instituto de Investigaciones Sanitarias de Santiago (IDIS). Santiago de Compostela, Spain; 6 Departamento de Estadística, Análisis Matemático y Optimización. Grupo Interdisciplinar de Bioestadística y Ciencia de Datos Biométricos (GRID-BDS), Universidad de Santiago de Compostela. Santiago de Compostela, Spain; 7 Subdirección de Información sobre Saúde e Epidemioloxía. Dirección Xeral de Saúde Pública, Consellería de Sanidade, Xunta de Galicia. Santiago de Compostela, Spain

**Keywords:** COVID-19, prediction model, severity, hospitalization, admission to ICU, death

## Abstract

**Background:**

The prognosis of patients with COVID-19 infection is uncertain. We derived and validated a new risk model for predicting progression to disease severity, hospitalization, admission to intensive care unit (ICU) and mortality in patients with COVID-19 infection (Gal-COVID-19 scores).

**Methods:**

This is a retrospective cohort study of patients with COVID-19 infection confirmed by reverse transcription polymerase chain reaction (RT-PCR) in Galicia, Spain. Data were extracted from electronic health records of patients, including age, sex and comorbidities according to International Classification of Primary Care codes (ICPC-2). Logistic regression models were used to estimate the probability of disease severity. Calibration and discrimination were evaluated to assess model performance.

**Results:**

The incidence of infection was 0.39% (10 454 patients). A total of 2492 patients (23.8%) required hospitalization, 284 (2.7%) were admitted to the ICU and 544 (5.2%) died. The variables included in the models to predict severity included age, gender and chronic comorbidities such as cardiovascular disease, diabetes, obesity, hypertension, chronic obstructive pulmonary disease, asthma, liver disease, chronic kidney disease and haematological cancer. The models demonstrated a fair–good fit for predicting hospitalization {AUC [area under the receiver operating characteristics (ROC) curve] 0.77 [95% confidence interval (CI) 0.76, 0.78]}, admission to ICU [AUC 0.83 (95%CI 0.81, 0.85)] and death [AUC 0.89 (95%CI 0.88, 0.90)].

**Conclusions:**

The Gal-COVID-19 scores provide risk estimates for predicting severity in COVID-19 patients. The ability to predict disease severity may help clinicians prioritize high-risk patients and facilitate the decision making of health authorities.

**Key Messages**

- The clinical manifestations of COVID-19 range from asymptomatic infection (80%) to pneumonia (15–20%), which may progress to acute respiratory distress syndrome, multiorganic failure and, ultimately, death (1–5%). The course of these patients is unknown.
- The early accurate identification of COVID-19 patients at a higher risk of developing severe disease may help clinicians prioritize high-risk patients and facilitate the decision making of health authorities.
- This study shows that a prognostic model based on clinical data routinely recorded by general practitioners in electronic health records can predict COVID-19 progression to severe disease, hospitalization, critical care or death.

## Introduction

In December 2019, China reported to the World Health Organization (WHO) several cases of pneumonia of unknown origin in Wuhan, in the province of Hubei.^1^ These cases were later confirmed to be caused by a novel coronavirus, the severe acute respiratory syndrome coronavirus 2 (SARS-CoV-2), which was renamed coronavirus disease 2019 (COVID-19).^2^ The disease rapidly spread to most countries in the world. By May 31, 5.9 million people had become infected and 367 166 had died.^3^

The clinical manifestations range from asymptomatic infection to pneumonia, which can progress to acute respiratory distress syndrome, multiorganic failure and, ultimately, death.^1^^,^^4–6^ About 80% of reported cases have mild symptoms, but 15–20% will progress to severe pneumonia that will cause death to 1–5% of patients. According to predictive models,^7^ age and the presence of some particular comorbidities (hypertension, cardiorespiratory disease or diabetes)^5^ are associated with a higher risk for disease progression.

No specific therapies or vaccines have yet been developed to prevent or reduce the risk of developing complications of COVID-19. For health authorities to be able to allocate the resources necessary in each health district, it is crucial that COVID-19 patients at a higher risk of developing severe disease [i.e. hospitalization, ICU (intensive care unit) admission or death]^8–10^ are identified early and accurately.

The purpose of this study was to develop and validate a prognostic model to identify patients with COVID-19 infection at a higher risk of hospitalization, ICU admission and death, based on their age, gender, comorbidities and geographic place of residence. These data are available in the electronic health records (EHR) of Primary Care centres and are classified in accordance with the International Classification of Primary Care (ICPC-2).

## Methods

### Source of data

A retrospective cohort study was performed of patients diagnosed with COVID-19 in any of its clinical forms in Galicia, Spain, from 6 March 2020, when the first case in the region was reported, to 7 May 2020. Galicia is a region in the northwest of Spain [(area: 29 574.4 km^2^; population: 2 700 441 inhabitants (1 303 453 males; population density: 91.3 inhabitants/km^**2**^)] with a mean age of 47.2 years and 515 488 people >70 years old (19.1% of the total population).

Data were collected from the Galician Health Service (*Servizo Galego de Saúde*, SERGAS) database, which contains longitudinal data of the population in Galicia. The SERGAS database is based on data from 63 databases of healthcare services serving >95% of the population^11^ including public healthcare services, hospitals, primary care centres, pharmacies, emergency services, state-subsidized health entities and stakeholders. Epidemiological data were obtained using ICPC-2 from EHR of Primary Care centres using an automated technique.

The study was conducted in accordance with the guidelines of the Declaration of Helsinki and the principles of good clinical practice and was approved by the Institutional Review Board (IRB) of the Galician Health Service on 3 April 2020 (#2020/194). Informed consent forms were waived by the IRB.

### Definitions

A confirmed case of COVID-19 was defined as a positive reverse transcription polymerase chain reaction (RT-PCR) test on samples obtained from nasal or throat swabs performed in accordance with WHO protocol.^12^ RT-PCR was performed in people with symptoms consistent with COVID-19 (i.e. fever, chills, severe tiredness, sore throat, cough, shortness of breath, headache, anosmia or ageusia, and nausea, vomiting or diarrhoea), or contact with suspected or confirmed cases. Only laboratory-confirmed cases are registered in a single database and were considered for analysis.

Patients with uncomplicated disease, but with a oxygen saturation (SaO_2_)  > 95% and a respiratory rate <25 breaths/min, all considered as low-risk (<60 years of age and without comorbidities), and high-risk patients (>60 years and with comorbidities), were monitored as follows. (i) At home by the TELEA system, a home monitoring platform for monitoring respiratory and heart rate, temperature and SaO_2_.^13^ (ii) Patients without internet connection at home were monitored via 2–3 telephone calls daily. If the clinical status of the patient deteriorated, a physician contacted them to decide whether hospitalization was required or not. (iii) Previously-institutionalized patients or those without enough assistance at home were transferred to a socio-health centre adapted as a hospital. All patients diagnosed with COVID-19 pneumonia were hospitalized. Pneumonia was defined as an acute respiratory disorder characterized by cough, at least a novel condensation on thoracic X-ray, and a fever of four or more days of duration, or dyspnea/tachypnea.^14^ COVID-19 was considered severe and the patient was a candidate for ICU admission if they required mechanical ventilation or had a fraction of inspired oxygen of ≥60%.^15^

Patient’s total comorbid burden was determined by the Charlson Comorbidity Index, which predicts the 10-year life expectancy of patients with multiple comorbidities.^16^

### Outcomes

We focused on three key outcomes: hospitalization, ICU admission and death of any cause after RT-PCR diagnosis in the study period.

### Predictors

Based on a review of existing literature to identify comorbidities associated with COVID-19 prognosis,^5^^,^^9^ the following data were extracted from the EHR of each patient: age, sex and ICPC-2 comorbidities [allergy, lymphoma/leukaemia, acquired immune deficiency syndrome (AIDS), malignant neoplasm, peptic ulcer, chronic enteritis, liver disease, ischaemic heart disease, heart failure, atrial fibrillation, heart valve disease, hypertension, cerebrovascular disease, peripheral vascular disease, rheumatoid arthritis, alcohol and drug abuse, tobacco abuse, dementia, psychosis, chronic obstructive pulmonary disease, asthma, malignant neoplasm of skin, psoriasis, obesity, diabetes, lipid disorder and chronic kidney disease]. An individual was considered to have any of these conditions if they had suffered it at some point of their life. A detailed description of comorbidities and their corresponding codes is available in Supplementary Table 1, available as Supplementary data at *IJE* online. 

### Statistical analysis

We used a random sample of 70% to derive the multivariable logistic regression models, and their performance, and the remaining 30% for validation. First, in the derivation sample, all predictors described above were included in the models to estimate the probability of hospital admission or evolving in a critical case or death. Final models included age, sex and a combination of comorbidities. Beginning with a model containing all potential covariates, the variable with the least significant *P* value was removed and tested using the likelihood-ratio test until all variables left in the model significantly (at alpha = 0.05) contributed to the model.

Results are presented as odds ratio (OR) with 95% confidence intervals (CIs). The Nagelkerke *R*^2^ was used to calculate the proportion of the explained variance of clinical outcomes by the selected predictors. The different aspects of model performance were studied, including calibration and discrimination. Calibration was assessed using the Brier score and by plotting the non-parametric estimate of the association between the observed frequencies and the predicted probabilities.^17^ The receiver operating characteristics (ROC) curves [and the corresponding area under the ROC curve (AUC)] were calculated to test for discrimination. To correct optimism, internal validation was performed for each model using the bootstrap procedure with 500 bootstrapped samples.^17^ The final models were selected to derive scores for clinical use (Gal-COVID-19 score), and nomograms were created. Criteria for this selection included both discriminant ability (defined by the AUC) and model simplicity. Finally, the coefficients (scores) derived from the derivation cohort were also validated on the validation cohort.

To verify the robustness of the models, additional regression analyses were performed to predict admission to ICU and mortality in those patients who had completed the course of the disease.

All statistical analyses were carried out in R version 3.5.1 using the packages BayesX and rms. These packages are freely available at http://cran.r-project.org. The analysis conforms to the reporting standards of TRIPOD.^18^

## Results

A total of 10 454 subjects [4172 men (40%); mean age 58 years] acquired the disease, which accounts for 0.39% of the population. Of them, 2492 cases (23.8%) required hospitalization, 284 (2.7%) were admitted to the ICU and 544 (5.2%) died [of whom 154 (28.3%) had not been hospitalized].

The median length of stay was 19 days (interquartile range 7, 38). At the end of the study, 291 (11.6%) hospitalized patients had not yet been discharged, had been transferred to the ICU or died. The patients who were still hospitalized were older and had fewer comorbidities than hospitalized patients who had been discharged or died. The median ICU stay time was 15 days (interquartile range 3, 28). Of the patients admitted to ICU, 43 (15.1%) had not yet been discharged or died.

**Figure 1:**
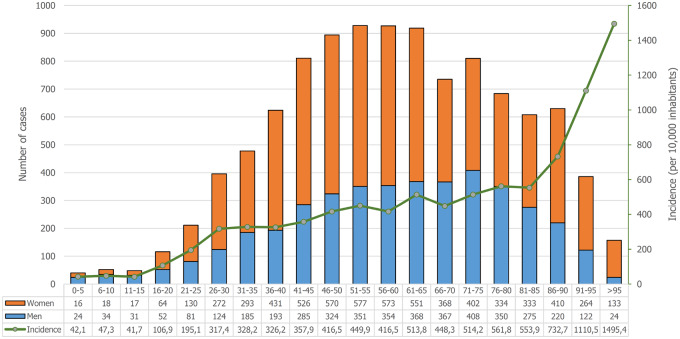
Age and gender distributions of patients with SARS-CoV-2 infection (crude cases and incidence).

**Figure 2:**
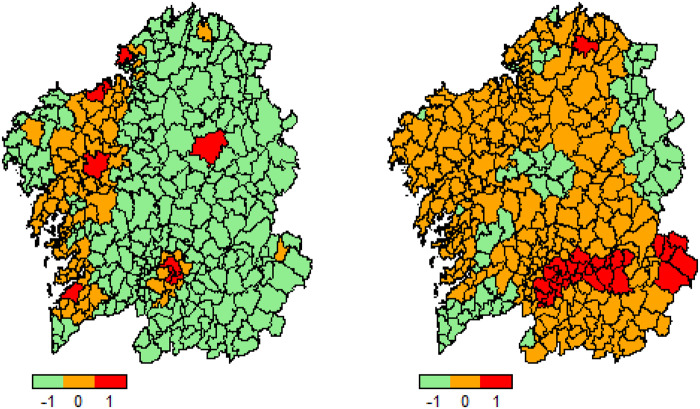
Distributions of patients with SARS-CoV-2 infection by municipality. Cases (left) and incidence (right). Low incidence is shown in green whereas high incidence is displayed in red.

Figure 1 shows the distribution of COVID-19 by age and gender and the incidence of the disease by age group. Supplementary Table 2, available as Supplementary data at *IJE* online, shows the total population of Galicia, the number of COVID-19 positives and their distribution by age and gender. Figure 2 gives the distribution of all laboratory-confirmed cases of COVID-19 reported by municipalities in Galicia in accordance with official statistics, expressed as absolute values and as percentages of the population. The highest incidence was observed in municipalities located in the southeast of the region, which coincides with the highway that connects Madrid with Galicia.

Table 1 displays the demographic characteristics (age and gender) and comorbidities of patients. Severity of disease increases with age and frequency of comorbidities, and is higher in men than in women (Supplementary Figure 1, available as Supplementary data at *IJE* online). Notably, the patients who died out of hospital were the ones with a more advanced age and a higher prevalence of chronic diseases such as dementia, dependence and immobilization. The median Charlson Comorbidity Index Score was 2 (interquartile range 1, 4).

**Table 1:** Clinical characteristics of the study patients: demographics, comorbidities and outcomes; figures are means (SD) or *n* (%).

Characteristics	Global (*n *=* *10 454)	Non-hospitalized (*n* = 7962) Alive (*n* = 7808) Death (*n* = 154)		Hospitalized (*n* = 2492) Alive (*n* = 2102) Death (*n* = 390)		ICU (*n *=* *284)	All deaths (*n *=* *544)
Age, years	58.0 (20.0)	53.6 (19.3)	85.7 (10.3)	68.3 (16.1)	80.4 (1.6)	65.8 (11.0)	81.9 (10.7)
Male sex	4172 (39.9)	2780 (35.6)	68 (44.2)	1085 (51.6)	239 (61.3)	196 (69.0)	307 (56.4)
Immobilized	53 (0.5)	25 (0.3)	6 (3.9)	10 (0.5)	12 (3.1)	0 (0.0)	18 (3.3)
Dependence	132 (1.3)	79 (1.0)	11 (7.1)	20 (1.0)	22 (5.6)	0 (0.0)	33 (6.1)
Allergy	311 (3.0)	240 (3.1)	3 (1.9)	59 (2.8)	9 (2.3)	14 (4.9)	12 (2.2)
Lymphoma/leukaemia	34 (0.3)	19 (0.2)	0 (0.0)	7 (0.3)	8 (2.1)	1 (0.4)	8 (1.5)
AIDS	9 (0.1)	7 (0.1)	0 (0.0)	2 (0.1)	0 (0.0)	0 (0.0)	0 (0.0)
Malignant neoplasm	238 (2.3)	131 (1.7)	8 (5.2)	81 (3.9)	18 (4.6)	7 (2.5)	26 (4.8)
Peptic ulcer	12 (0.1)	8 (0.1)	0 (0.0)	4 (0.2)	0 (0.0)	1 (0.4)	0 (0.0)
Chronic enteritis	32 (0.3)	18 (0.2)	1 (0.6)	11 (0.5)	2 (0.5)	1 (0.4)	3 (0.6)
Liver disease	149 (1.4)	89 (1.1)	4 (2.6)	42 (2.0)	14 (3.6)	18 (6.3)	18 (3.3)
Ischaemic heart disease	227 (2.2)	95 (1.2)	11 (7.1)	81 (3.9)	40 (10.3)	20 (7.0)	51 (9.4)
Heart failure	98 (0.9)	29 (0.4)	6 (3.9)	40 (1.9)	23 (5.9)	1 (0.4)	29 (5.3)
Atrial fibrillation	204 (2.0)	90 (1.2)	6 (3.9)	77 (3.7)	31 (7.9)	10 (3.5)	37 (6.8)
Heart valve disease	83 (0.8)	35 (0.4)	1 (0.6)	32 (1.5)	15 (3.8)	3 (1.1)	16 (2.9)
Hypertension	1457 (13.9)	749 (9.6)	51 (33.1)	503 (23.9)	154 (39.5)	87 (30.6)	205 (37.7)
Cerebrovascular disease	156 (1.5)	80 (1.0)	9 (6.1)	41 (1.9)	26 (6.7)	6 (2.1)	35 (6.4)
Peripheral vascular disease	82 (0.8)	37 (0.5)	2 (1.3)	28 (1.3)	15 (3.8)	5 (1.8)	17 (3.1)
Rheumatoid arthritis	39 (0.4)	20 (0.3)	0 (0.0)	15 (0.7)	4 (1.0)	0 (0.0)	4 (0.7)
Alcohol/drug abuse	66 (0.6)	35 (0.4)	2 (1.3)	20 (1.0)	9 (2.3)	3 (1.1)	11 (2.0)
Tobacco abuse	258 (2.3)	161 (2.1)	3 (1.9)	75 (3.6)	19 (4.9)	18 (6.3)	22 (2.0)
Dementia	194 (1.9)	116 (1.5)	25 (16.2)	31 (1.5)	22 (5.6)	1 (0.4)	47 (8.6)
Psychosis	55 (0.5)	34 (0.4)	2 (1.3)	15 (0.7)	4 (1.0)	0 (0.0)	6 (1.1)
COPD^a^	180 (1.7)	69 (0.9)	5 (3.2)	71 (3.4)	35 (9.0)	11 (3.9)	40 (7.4)
Asthma	288 (2.8)	184 (2.4)	1 (0.6)	91 (4.3)	12 (3.1)	14 (4.9)	13 (2.4)
Malignant neoplasm of skin	46 (0.4)	25 (0.3)	2 (1.3)	14 (0.7)	5 (1.3)	1 (0.4)	7 (1.3)
Psoriasis	62 (0.8)	51 (0.7)	2 (1.3)	15 (0.7)	6 (1.5)	6 (2.1)	8 (1.5)
Obesity	678 (6.5)	385 (4.9)	11 (7.1)	229 (10.9)	53 (13.6)	53 (18.7)	64 (11.8)
Diabetes	619 (5.9)	278 (3.6)	21 (13.6)	232 (11.0)	88 (22.6)	52 (18.3)	109 (20.0)
Lipid disorder	1490 (14.3)	852 (10.9)	35 (22.7)	482 (22.9)	121 (31.0)	83 (29.2)	156 (28.7)
Chronic kidney disease	101 (1.0)	41 (0.5)	4 (2.6)	30 (1.4)	26 (6.7)	5 (1.8)	30 (5.5)
Charlson Index							
0	2078 (19.9)	1957 (25.1)	2 (1.3)	118 (5.6)	1 (0.3)	8 (2.8)	3 (0.6)
1–2	3445 (33.0)	3002 (38.4)	1 (0.6)	424 (20.2)	18 (4.6)	55 (19.4)	19 (3.5)
3–4	3875 (37.1)	2376 (30.4)	91 (59.1)	1197 (56.9)	211 (54.1)	163 (57.4)	302 (55.5)
≥5	1056 (10.1)	473 (6.1)	60 (39.0)	473 (17.3)	160 (41.0)	58 (20.4)	220 (40.4)

a COPD, chronic obstructive pulmonary disease.

### Predictors in the derivation sample and performance of the models

The number of hospitalizations in the derivation cohort was 1745 [incidence (5%CI): 23.8 (2.9, 24.8) hospitalizations per 100 COVID-19 patients; Supplementary Table 3, available as Supplementary data at *IJE* online]. The variables included in the model to predict hospitalization included age, gender, dependence, heart failure, hypertension, rheumatoid arthritis, tobacco abuse, dementia, chronic obstructive pulmonary disease, asthma, obesity and diabetes (Table 2). The AUC obtained for this model was 0.77 (95%CI: 0.76, 0.78). The AUC corrected by bootstrapping was 0.77. Nagelkerke *R*^2^ was 0.25.

**Table 2:** Multivariable logistic regression models predicting hospitalization, intensive care unit admission and death

	Hospitalization		ICU Admission		Death	
	Coefficient (SE)	OR (95%CI)	Coefficient (SE)	OR (95%CI)	Coefficient (SE)	OR (95%CI)
Age, years	1.902 (0.081)	6.70 (5.71, 7.86)	1.693 (0.211)	5.44 (3.59, 8.22)	3.019 (0.374)	20.5 (9.83, 42.6)
Male sex	0.673 (0.051)	1.96 (1.77, 2.17)	1.020 (0.134)	2.77 (2.13, 3.60)	0.859 (0.102)	2.36 (1.93, 2.88)
Dependence	−0.471 (0.204)	0.62 (0.42, 0.93)				
Lymphoma/leukaemia					1.449 (0.489)	4.26 (1.63, 11.1)
Liver disease			0.996 (0.279)	2.71 (1.57, 4.68)		
Ischaemic heart disease					0.478 (0.186)	1.61 (1.20, 2.33)
Heart failure	0.731 (0.227)	2.08 (1.33, 3.24)				
Hypertension	0.229 (0.073)	1.26 (1.09, 1.45)				
Rheumatoid arthritis	0.727 (0.354)	2.07 (1.03, 4.14)				
Tobacco abuse	0.362 (0.152)	1.44 (1.06, 1.94)				
Dementia					0.558 (0.187)	1.75 (1.21, 2.52)
COPD^a^	0.425 (0.167)	1.53 (1.10, 2.12)			0533 (0.206)	1.70 (1.14, 2.55)
Asthma	0.732 (0.143)	2.08 (1.57, 2.75)				
Obesity	0.268 (0.098)	1.31 (1.08, 1.58)	0.625 (0.179)	1.87 (1.32, 2.65)		
Diabetes	0.357 (0.098)	1.43 (1.18, 1.73)	0.475 (0.184)	1.61 (1.12, 2.31)	0.584 (0.132)	1.79 (1.38, 2.32)
Chronic kidney disease					0.820 (0.249)	2.27 (1.39, 3.70)

a COPD, chronic obstructive pulmonary disease..

The number of ICU admissions was 193 [incidence (95%CI): 2.6% (2.3, 3.0)]. The model to predict ICU admission included age, gender, liver disease, obesity and diabetes (Table 2). The AUC obtained for this model was 0.83 (95%CI: 0.81, 0.85). The AUC corrected was 0.82. Nagelkerke *R*^2^ was 0.17.

The number of deaths was 384 [incidence (95%CI): 5.2% (4.7, 5.7)]. The model to predict death included age, gender, haematological cancer, ischaemic heart disease, dementia, chronic obstructive pulmonary disease, diabetes and chronic liver disease (Table 2). The AUC obtained for this model was 0.89 (95%CI: 0.88, 0.90). The AUC corrected was 0.89. Nagelkerke *R*^2^ was 0.31.

### Model validation and sensitivity analysis

Hospitalization was observed in 747 participants in the validation cohort [incidence (95%CI): 23.8 (22.3, 25.3) hospitalizations per 100 COVID-19 person; Supplementary Table 3, available as Supplementary data at *IJE* online). The AUC of the validation dataset cohort was 0.77 (95%CI: 0.75, 0.79) with the Gal-COVID-19 model and 0.73 (95%CI: 0.71, 0.75) with the Charlson index score model. Nagelkerke *R*^2^ was 0.24.

ICU admission was observed in 91 participants [incidence (95%CI): 2.9% (2.3, 3.5)]. The AUC of the validation dataset cohort was 0.83 (95%CI: 0.79, 0.87) with the Gal-COVID-19 model and 0.70 (95%CI: 0.65, 0.74) with the Charlson score model. Nagelkerke *R*^2^ was 0.17.

Death was observed in 160 participants [incidence (95%CI): 5.1% (4.3, 5.9)]. The AUC of the validation dataset cohort was 0.86 (95%CI: 0.84, 0.89) with the Gal-COVID-19 model and 0.83 (95%CI: 0.81, 0.86) with the Charlson score model.

The Brier scores of Gal-COVID-19 and Charlson index in the validation cohort were 0.150 and 0.157, respectively for predicting hospitalization, 0.025 and 0.026, respectively for predicting ICU admission, and 0.043 and 0.046, respectively for predicting death.

The Nagelkerke *R*^2^ of Gal-COVID-19 and Charlson index in the validation samples were 0.24 and 0.19 for predicting hospitalization, 0.17 and 0.004, respectively, for predicting ICU admission, and 0.25 and 0.23, respectively, for predicting death.

**Figure 3:**
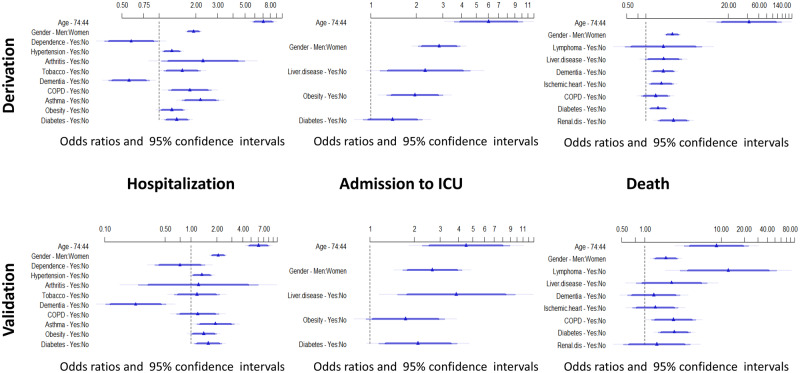
Forest plots showing the adjusted ORs for hospitalization, ICU admission and death. Upper panel derivation cohort. Lower panel validation cohort. The ORs come from logistic models using age, gender and comorbidities as predictors.

We fitted regression models with the validation data to estimate the coefficients for each risk factor. None of these coefficients differed significantly from those in the derivation sample, either in hospitalization, ICU admission or death (Figure 3).

**Figure 4:**
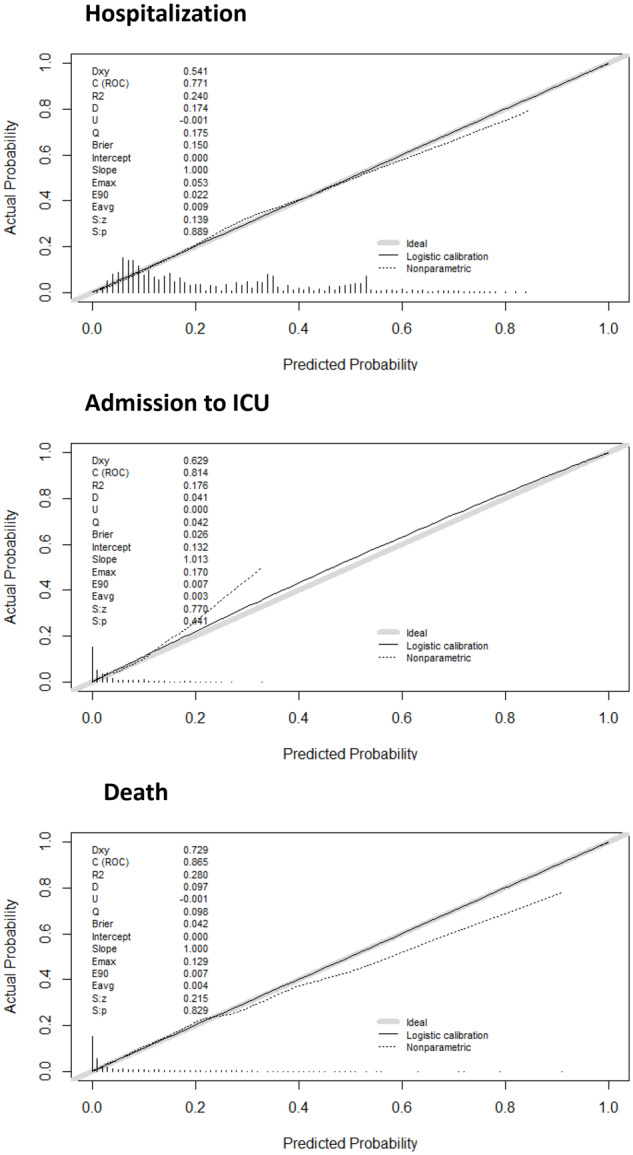
Calibration plots of the final models for predicting hospitalization, ICU admission and death in the derivation cohort. The dotted line shows the actual relation between observed outcomes and predicted risks; the solid line shows the smoothed relation. Ideally, these lines equal the dashed diagonal line that represents perfect calibration.

Participants in the validation dataset were divided into groups of predicted probabilities according to the distribution of the Gal-COVID-19 scores for risk of hospitalization, ICU admission and death. Overall, the rates of incidence of observed hospitalizations were similar to those predicted by Gal-COVID-19 scores in the groups of predicted risk in the validation dataset. There was slight under- and over-estimation of risk amongst highest risk strata for ICU admission and death, respectively (Figure 4).

Figures in the supplementary material illustrate a method to estimate the risk of progression to hospitalization, ICU admission and death based on an overall score calculated by the sum of the individual scores obtained in the variables of the model (Supplementary Figures 2, 3 and 4, respectively, available as Supplementary data at *IJE* online).

Table 3 shows the individual score of each of the predictors for predicting hospitalization, ICU admission and death. Supplementary material provides a spreadsheet in excel format (Supplementary Material 2, GalCOVID-19.Score.xls, available as Supplementary data at *IJE* online) for estimating risk of hospitalization, ICU admission and death.

**Table 3:** Scores for hospitalization, ICU admission and death^a^

Comorbidities	Points	Age, years	Points	Total	Risk
				Hospitalization	
Men	20	10	9	39	0.02
Non-dependence	14	20	18	66	0.05
Heart failure	21	30	27	88	0.10
Rheumatoid arthritis	21	40	36	102	0.15
Tobacco abuse	11	50	47	112	0.20
Non dementia	22	60	65	120	0.25
COPD^b^	12	70	89	128	0.30
Asthma	21	80	100	141	0.40
Diabetes	10	90	97	153	0.50
		100	93	185	0.75
	
				ICU admission	
Men	9	10	51	87	0.01
Liver disease	9	20	62	101	0.05
Obesity	5	30	73	108	0.10
Diabetes	4	40	82	115	0.20
		50	86	117	0.25
		60	91	120	0.30
		70	100	123	0.40
		80	89	127	0.50
		90	61		
		100	31		
	
				Death	
Men	8	10	10	59	0.01
Lymphoma/leukaemia	13	20	30	75	0.05
Liver disease	6	30	31	82	0.10
Ischaemic heart disease	4	40	41	86	0.15
Dementia	5	50	49	89	0.20
COPD	5	60	58	92	0.25
Diabetes	5	70	68	102	0.50
Chronic kidney disease	8	80	78	112	0.75
		90	85		
		100	93		

a Enter the scores for gender and comorbidities in the left column and the patient’s age in the middle column. Sum the points obtained for each of the predictors. Enter the total score and the associated risk for hospitalization, admission and death in the right column. For example, a 60-year-old woman with asthma and diabetes with any other comorbidity, would sum 132 points [(age = 60 years, 65 points) + (dependence = no, 14 points) + (dementia = no, 22 points) + (asthma = yes, 21 points) + (diabetes = yes, 10 points)]. The risk for hospitalization would be >30%. Likewise, proceed to estimate the risk for ICU admission and death.

b COPD, Chronic obstructive pulmonary disease.

**Figure 5:**
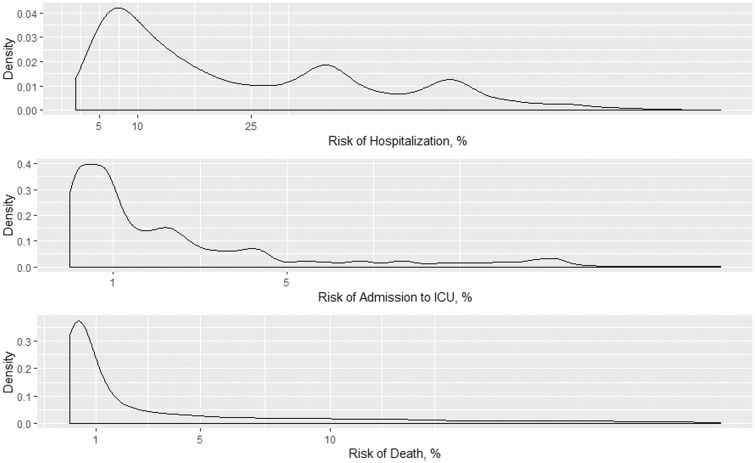
Density distributions of risk hospitalization (top), admission to ICU (middle) and death (bottom) in the entire cohort.

The distribution of risk of hospitalization, ICU admission and death is shown in Figure 5. The risk thresholds of 5, 10 and 25% for hospitalization accounted for 7.4, 30.8 and 59.7% of the COVID-19 population, respectively. For predicting ICU admission, the risk thresholds of 1 and 5% accounted for 46.5 and 84.8% of the COVID-19 population, respectively. For predicting death, the risk thresholds of 1 and 5% accounted for 50.3 and 71.6% of the COVID-19 population, respectively.

Of the hospitalized patients, 291 (11.6%) had not yet been discharged, admitted to the ICU or died. No differences in estimates were found between analyses performed to estimate the risk of ICU admission or death, respectively, on all patients and on those who had completed the course of the disease (Supplementary Tables 4 and 5, available as Supplementary data at *IJE* online).

## Discussion

A population-based study was performed to derive and validate new risk models for predicting hospitalization, ICU admission and mortality in patients with COVID-19 infection. Data were extracted from EHR generated by general practitioners as part of their routine practice. The results of this study are of relevance as the course of COVID-19 is unknown and may cause death. In most cases, the disease can be controlled by closely monitoring its course. However, critical patients require hospitalization, the administration of aggressive treatments and critical care.

In Galicia, 0.39% of the population tested positive for COVID-19 in RT-PCR tests. A seroepidemiological population-based survey sponsored by the Spanish Government (ENE-Covid19) revealed that 2.1% of this population was positive for COVID-19.^19^ This inconsistency may be due to the fact that one in three infections seems to be asymptomatic, while a substantial number of symptomatic cases were not tested. It may affect the evaluation of the extent of the epidemic but not the models for predicting outcomes.

In total, 28.3% of patients who died (154 patients) had not been hospitalized. This may be explained by the fact that previously institutionalized patients, who had an older age, disabilities, and chronic terminal illnesses, were transferred to socio-health centres adapted as a hospital. Unlike other regions in Spain, the COVID-19 pandemic did not lead to shortages of hospitalization and ICU facilities. Since there were no clear guidelines in this regard, the decision for hospital admission and ICU admission was made following the primary role of beneficence and nonmaleficence in resource allocation according to pre-COVID criteria.

This predictive model is based on age, gender, presence of chronic diseases and risk factors (i.e. cardiovascular disease, neoplasm, diabetes, chronic obstructive pulmonary disease, obesity, hypertension, liver disease, chronic kidney disease). These factors have been demonstrated to be powerful predictors of progression and mortality^4^^,^^6^^,^^20–22^ and are routinely recorded in primary care. The results of this study confirm that age is a risk factor of hospitalization, ICU admission and death. The effect of age on T- and B-cell function and excessive production of type 2 cytokines probably reduce control of viral replication and result in a prolonged inflammatory response, which facilitates disease progression.^23^ In most studies, the disease had a higher prevalence in men,^1^^,^^4–7^^,^^24^ whereas in our study the disease was more frequent in women (60.1% of women). This may be due to the fact that studies generally include critical patients and, in this subgroup of patients, men are twice as likely as women to require hospitalization (OR 1.96, 95%CI 1.77, 2.17), need intensive care (OR 2.77, 95%CI 2.13, 3.60) or die (OR 2.36, 95%CI 1.93, 2.88). Likewise, there is consistent evidence that severe patients usually have more comorbidities than patients with mild disease.^4^^,^^6^^,^^20^ Thus, pneumonia may increase the risk of cardiovascular events,^25^ and other diseases such as arterial hypertension,^26^ diabetes^27^^,^^28^ and obesity^29^ may contribute to a poorer prognosis of COVID-19 infection.

The findings of this study have relevant clinical implications. Our prediction models may be useful to predict disease severity in patients with COVID-19 infection in primary care or community-based settings. This tool may help clinicians prioritize high-risk patients and decide whether they need to be referred to a hospital, where a diagnosis and appropriate treatment for the characteristics of the patient will be established. In addition, this predictive model identifies patients with severe disease who will probably need intensive care, and provides key information to the patient and their family on disease prognosis. In addition, these scores make it possible to establish risk levels, even arbitrarily, which may be useful to guide decision-making.

This study has two strengths: first, it is a population-based study that accounts for virtually the totality of cases of COVID-19 diagnosed in a well-defined region (Galicia, Spain). Second, this study is based on a high-quality, internally-validated database of EHR that provides a large sample, reflects real-life conditions and includes individuals who are not generally recruited in cohort studies. There are other studies investigating the association between comorbidities and disease progression, but most have been conducted in hospitalized patients but not in the general population. In population-based studies such as the UK-Biobank Cohort, subjects who had been previously evaluated were followed-up until confirmation of COVID-19 infection or COVID-19-related admission.^30^^,^^31^ Similar results are also found in an international study from six countries to develop and validate a risk score analyzing electronic medical records. In the development database their authors sample 150 000 patients with influenza or flu-like symptoms.^32^ In addition, a recent systematic literature review revealed ten prognostic models for predicting mortality or progression to severe disease, but only one study involved patients from countries other than China, and all studies had been categorized as being at a high risk of bias.^33^

The study also has several limitations. First, since the model was developed based on a single population, the lack of external validation is a major limitation. In addition, the calibration results suggest that the model’s performance should be assessed and recalibrated when used in other populations. Further studies are needed to generalize the clinical value of this predictive model in other geographic areas. Second, disease classification systems (ICPC-2 in this case) may lead to underdiagnosis.^34^ Third, outcomes such as discharge disposition or death were not available for patients still in hospital at the end of the study, because they had not completed their hospital course. Although no differences were found in the estimates between analyses performed on the totality of patients and those who had completed the course of the disease, the probability of death or ICU admission may have been underestimated.

## Conclusion

Our results provide evidence that age, gender and comorbidities, which are routinely recorded by general practitioners in EHR, may be useful to predict COVID-19 severity, need for hospitalization or ICU admission and death. This information may help clinicians to prioritize high-risk patients and facilitate the adoption of the appropriate healthcare strategies.

## Supplementary data

Supplementary data are available at *IJE* online.

## Funding

Instituto de Salud Carlos III (ISCIII), Spain, Grant/Award Number: COV20/00404; Ministry of Economy and Competitiveness (SPAIN) cofinanded and the Fondo Europeo de Desarrollo Regional (FEDER).

## Data availability

The data underlying this article will be shared on reasonable request to the corresponding author.

## Supplementary Material

dyaa209_Supplementary_Data
