# Supplementary material for: Development and validation of a prognostic model based on comorbidities to predict COVID-19 severity: a population-based study
Source: Int J Epidemiol. 2020 Dec 8;50(1):64–74. doi: 10.1093/ije/dyaa209 (PMC7799114; doi:10.1093/ije/dyaa209)
Supplement: dyaa209_Supplementary_Data [file dyaa209_supplementary_data.zip › ije-2020-06-0983-File008.docx]

**Supplementary Table S1. Pathologies or symptoms in the International Classification of Primary Care-2 entered into the regression models for predicting hospitalization, admission to intensive care unit (ICU) and death.**

| **Symptoms / Morbidities** | **ICPC-2 Codes** |
| --- | --- |
| Dependence | Z62.01 |
| Immobilized | A28.01 |
| Allergy/conjunctivitis/rhinitis/dermatitis | A92, F71, R97, S87, S88 |
| Hodgkin´s disease/lymphoma/leukaemia | B72, B73, B74 |
| HIV-infection/AIDS | B90 |
| Malignant neoplasm | D74, D75, D76, D77, L71, N74, R84, R85, T71, U75, U76, U77, X75, X76, X77, Y77, Y78 |
| Peptic ulcer | D85, D86 |
| Chronic enteritis/ulcerative colitis | D94 |
| Liver disease | D97 |
| Ischemic heart disease | K74, K75, K76 |
| Heart failure | K77 |
| Atrial fibrillation/flutter | K78 |
| Heart valve disease | K83 |
| Hypertension | K86, K87 |
| Cerebrovascular disease | K89, K90, K91 |
| Peripheral vascular disease | K92 |
| Rheumatoid arthritis | L88 |
| Alcohol/drug abuse | P15, P18, P19 |
| Tobacco abuse | P17 |
| Dementia | P70 |
| Schizofrenia/psicosis | P72, P73, P98 |
| Chronic obstructive pulmonary disease | R95 |
| Asthma | R96 |
| Malignant neoplasm of skin | S77 |
| Psoriasis | S91 |
| Obesity | T82 |
| Diabetes | T89, T90, W85 |
| Lipid disorder | T93 |
| Chronic kidney disease | U99.01 |

ICU, intensive care unit; HIV, human immunodeficiency virus; AIDS, acquired immune deficiency syndrome

**Supplementary Table S2. Incidence of Covid-19 in the Autonomous Community of Galicia, Spain**

| **Age groups, years** | **Overall population** | | | **Men** | | | **Women** | | |
| --- | --- | --- | --- | --- | --- | --- | --- | --- | --- |
|  | **Total** | **Covid-19** | **Incidence** | **Total** | **Covid-19** | **Incidence** | **Total** | **Covid-19** | **Incidence** |
| [0-4) | 95,035 | 40 | 42.09 | 49,013 | 24 | 48.97 | 46,022 | 16 | 34.77 |
| [5-9) | 109,879 | 52 | 47.32 | 56,689 | 34 | 59.98 | 53,190 | 18 | 33.84 |
| [10, 14) | 115,177 | 48 | 41.67 | 59,048 | 31 | 52.50 | 56,129 | 17 | 30.29 |
| [15, 20) | 108,562 | 116 | 106.85 | 55,898 | 52 | 93.03 | 52,664 | 64 | 121.53 |
| [20, 25) | 108,128 | 211 | 195.14 | 55,230 | 81 | 146.66 | 52,898 | 130 | 245.76 |
| [25, 30) | 124,766 | 396 | 317.39 | 63,273 | 124 | 195.98 | 61,493 | 272 | 442.33 |
| [30, 35) | 145,626 | 478 | 328.24 | 72,933 | 185 | 253.66 | 72,693 | 293 | 403.06 |
| [35, 40) | 191,318 | 624 | 326.16 | 95,830 | 193 | 201.40 | 95,488 | 431 | 451.37 |
| [40, 45) | 226,585 | 811 | 357.92 | 113,020 | 285 | 252.17 | 113,565 | 526 | 463.17 |
| [45, 50) | 214,645 | 894 | 416.50 | 106,154 | 324 | 305.22 | 108,491 | 570 | 525.39 |
| [50, 55) | 206,263 | 928 | 449.91 | 101,157 | 351 | 346.99 | 105,106 | 577 | 548.97 |
| [55, 60) | 194,856 | 927 | 475.74 | 94,932 | 354 | 372.90 | 99,924 | 573 | 573.44 |
| [60, 65) | 178,875 | 919 | 513.77 | 86,077 | 368 | 427.52 | 92,798 | 551 | 593.76 |
| [65, 70) | 163,937 | 735 | 448.34 | 77,967 | 367 | 470.71 | 85,970 | 368 | 428.06 |
| [70, 75) | 157,521 | 810 | 514.22 | 72,506 | 408 | 562.71 | 85,015 | 402 | 472.86 |
| [75, 80) | 121,760 | 684 | 561.76 | 53,316 | 350 | 656.46 | 68,444 | 334 | 487.99 |
| [80, 85) | 109,761 | 608 | 553.93 | 43,868 | 275 | 626.88 | 65,893 | 333 | 505.36 |
| [85, 90) | 85,982 | 630 | 732.71 | 42,053 | 220 | 523.15 | 84,752 | 410 | 483.76 |
| [90, 95) | 34,758 | 386 | 1110.54 | 10,643 | 122 | 1146.29 | 24,115 | 264 | 1094.75 |
| [95, 107) | 10,499 | 157 | 1495.38 | 2,636 | 24 | 910.47 | 7,863 | 133 | 1691.47 |
| **Total** | **2,703,933** | **10,454** | **386.62** | **1,312,243** | **4,172** | **317.93** | **1,432,513** | **6,282** | **438.53** |

Incidence is expressed in cases per 100,000 inhabitants

**Supplementary Table S3. Comparison of participant characteristics and outcomes between derivation and validation datasets.**

| **Characteristics** | **Derivation sample** | **Validation sample** |
| --- | --- | --- |
|  | (*n*=7,317) | (*n*=3,137) |
| Hospitalizations | 1745 (23.8) | 747 (23.8) |
| ICU admissions | 193 (2.6) | 91 (2.9) |
| Exitus | 384 (5.2) | 160 (5.1) |
| Age, years | 58.1 (20.0) | 57.8 (20.0) |
| Male sex | 2944 (40.2) | 1228 (39.1) |
| Immobilized | 33 (0.4) | 20 (0.6) |
| Dependence | 86 (1.1) | 46 (1.4) |
| Allergy | 227 (3.1) | 84 (2.6) |
| Lymphoma/leukaemia | 24 (0.3) | 10 (0.3) |
| AIDS | 5 (0.1) | 4 (0.1) |
| Malignant neoplasm | 179 (2.4) | 59 (1.8) |
| Peptic ulcer | 8 (0.1) | 4 (0.1) |
| Chronic enteritis | 24 (0.3) | 8 (0.2) |
| Liver disease | 108 (1.4) | 41 (1.3) |
| Ischemic heart disease | 154 (2.1) | 73 (2.3) |
| Heart failure | 76 (1.0) | 22 (0.7) |
| Atrial fibrillation | 150 (2.0) | 54 (1.7) |
| Heart valve disease | 59 (0.8) | 24 (0.7) |
| Hypertension | 1034 (14.1) | 423 (13.5) |
| Cerebrovascular disease | 113 (1.5) | 39 (1.2) |
| Peripheral vascular disease | 59 (0.8) | 23 (0.7) |
| Rheumatoid arthritis | 32 (0.4) | 7 (0.2) |
| Alcohol/drug abuse | 49 (0.6) | 17 (0.5) |
| Tobacco abuse | 191 (2.6) | 67 (2.1) |
| Dementia | 146 (1.9) | 48 (1.5) |
| Psychosis | 36 (0.5) | 19 (0.6) |
| COPD | 120 (1.6) | 60 (1.9) |
| Asthma | 193 (2.6) | 95 (3.0) |
| Malignant neoplasm of skin | 33 (0.4) | 13 (0.4) |
| Psoriasis | 60 (0.8) | 22 (0.7) |
| Obesity | 472 (6.4) | 206 (6.5) |
| Diabetes | 444 (6.0) | 175 (5.5) |
| Lipid disorder | 1052 (14.3) | 438 (13.9) |
| Chronic kidney disease | 71 (0.9) | 30 (0.9) |

AIDS, acquired immune deficiency syndrome; COPD, chronic obstructive pulmonary disease; ICU, intensive care unit

**Supplementary Table S4.** Comparative regression analyses for predicting intensive care unit admission, in all patients and in those who completed the course of the disease (discharge or death).

|  | **All Patients** | | **Patients who completed the course of the disease** | |
| --- | --- | --- | --- | --- |
|  | **Coefficient (SE)** | **OR (95%CI)** | **Coefficient (SE)** | **OR (95%CI)** |
| Age, years | 1.693 (0.211) | 5.44 (3.59. 8.22) | 1.614 (0.257) | 5.02 (3.03, 8.32) |
| Men | 1.020 (0.279) | 2.77 (2.13, 3.60) | 1.101 (0.165) | 3.00 (2.17, 4.16) |
| Liver disease | 0.996 (0.279) | 2.71 (1.57, 4.68) | 1.005 (0.323) | 2.73 (1.44, 5.15) |
| Obesity | 0.625 (0.179) | 1.87 (1.32, 2.65) | 0.701 (0.213) | 2.01 (1.32, 3.06) |
| Diabetes | 0.475 (0.184) | 1.61 (1.12,2.31) | 0.547 (0.221) | 1.72 (1.12, 2.66) |

SE, standard error; OR (95%CI), Odds Ratio (95% Confidence Interval).

**Supplementary Table S5.** Comparative regression analyses for predicting death, in all patients and in those who completed the course of the disease (discharge or death)

|  | **All Patients** | | **Patients who completed the course of the disease** | |
| --- | --- | --- | --- | --- |
|  | **Coefficient (SE)** | **OR (95%CI)** | **Coefficient (SE)** | **OR (95%CI)** |
| Age, years | 3.019 (0.374) | 20.5 (9.83, 42.6) | 3.104 (0.393) | 22.3 (10.3, 48.2) |
| Men | 0.859 (0.102) | 2.36 (1.93, 2.88) | 0.909 (0.103) | 2.48 (2.02, 3.04) |
| Lymphoma | 1.449 (0.489) | 4.26 (1.63, 11.1) | 1.531 (0.494) | 4.62 (1.75, 12.2) |
| IHD | 0.478 (0.186) | 1.61 (1.20, 2.33) | 0.437 (0.187) | 1.54 (1.07, 2.23) |
| Dementia | 0.558 (0.187) | 1.75 (1.21, 2.52) | 0.487 (0.187) | 1.62 (1.12, 2.35) |
| COPD | 0.533 (0.206) | 1.70 (1.14, 2.55) | 0.459 (0.206) | 1.58 (1.05, 2.37) |
| Diabetes | 0.584 (0.132) | 1.79 (1.38, 2.32) | 0.528 (0.132) | 1.69 (1.30, 2.20) |
| Kidney disease | 0.820 (0.249) | 2.27 (1.39, 3.70) | 0.848 (0.251) | 2.33 (1.42, 3.82) |

COPD, chronic obstructive pulmonary disease; IHD, ischemic heart disease; Odds Ratio (95% Confidence Interval); SE, standard error; OR (95%CI)
