# Supplementary material for: Development and validation of a prognostic model based on comorbidities to predict COVID-19 severity: a population-based study
Source: Int J Epidemiol. 2020 Dec 8;50(1):64–74. doi: 10.1093/ije/dyaa209 (PMC7799114; doi:10.1093/ije/dyaa209)
Supplement: dyaa209_Supplementary_Data [file dyaa209_supplementary_data.zip › ije-2020-06-0983-File010.pptx]

## Slide 1
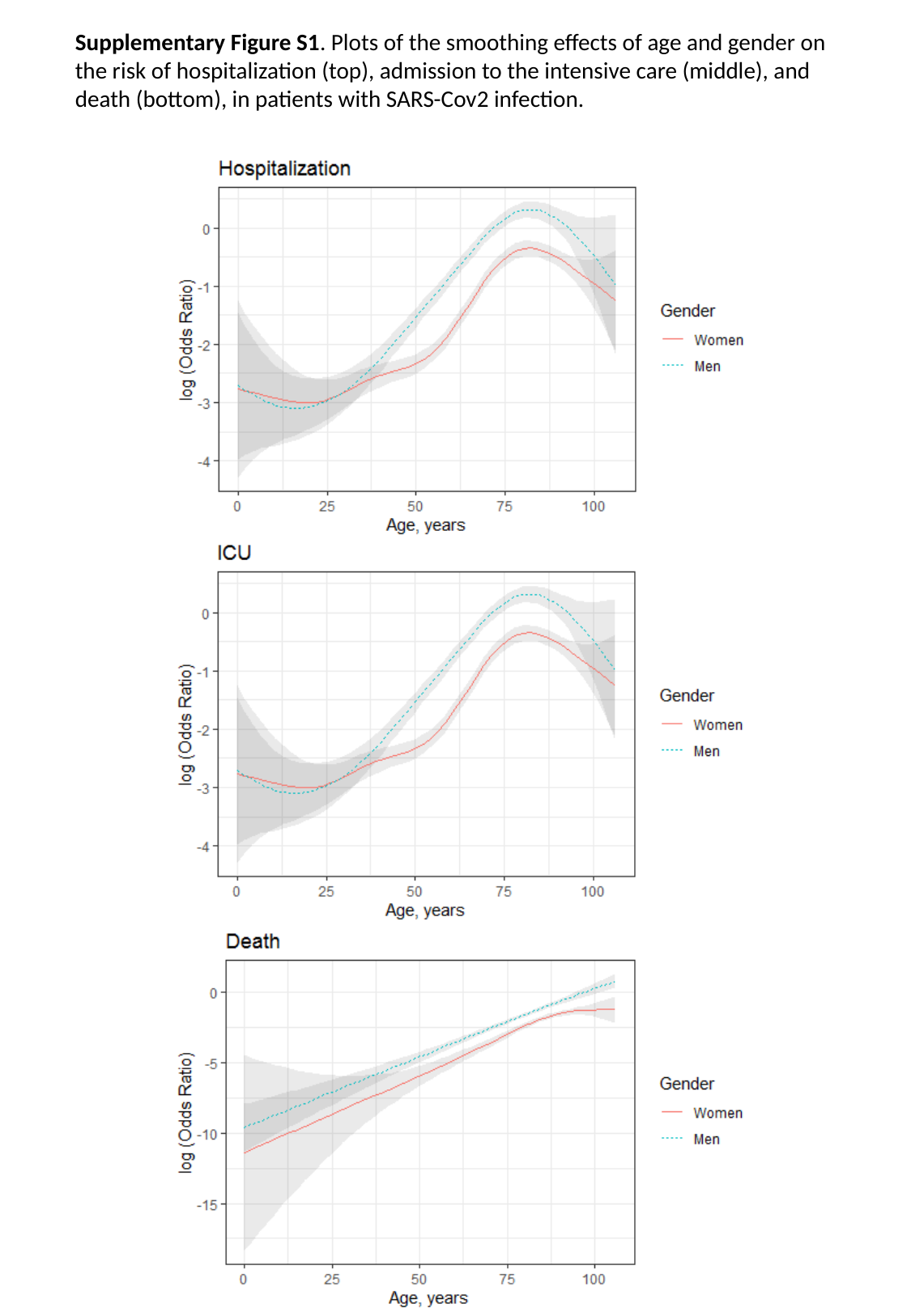

Supplementary Figure S1. Plots of the smoothing effects of age and gender on the risk of hospitalization (top), admission to the intensive care (middle), and death (bottom), in patients with SARS-Cov2 infection.

## Slide 2
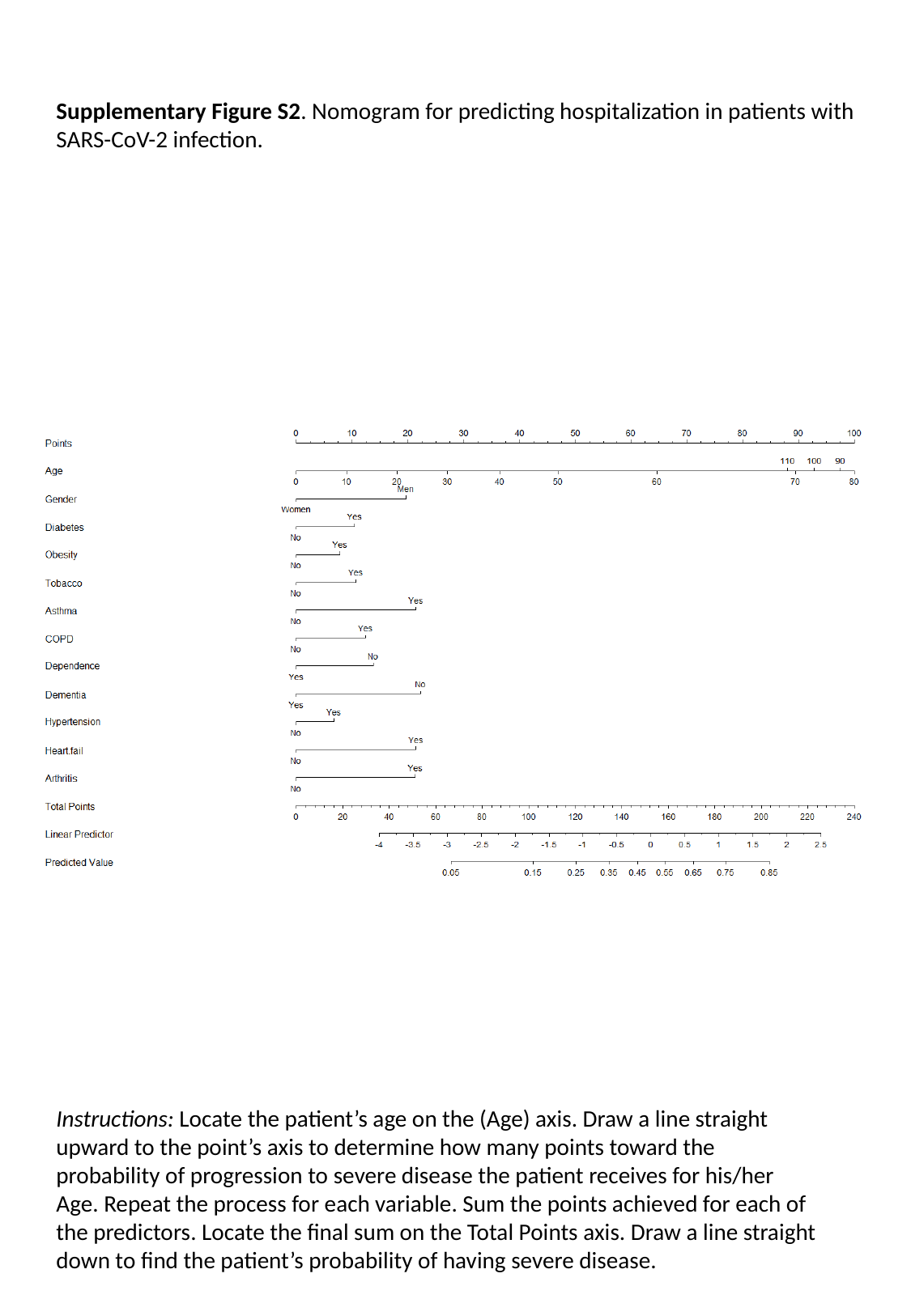

Supplementary Figure S2. Nomogram for predicting hospitalization in patients with SARS-CoV-2 infection.
Instructions: Locate the patient’s age on the (Age) axis. Draw a line straight upward to the point’s axis to determine how many points toward the probability of progression to severe disease the patient receives for his/her Age. Repeat the process for each variable. Sum the points achieved for each of the predictors. Locate the final sum on the Total Points axis. Draw a line straight down to find the patient’s probability of having severe disease.

## Slide 3
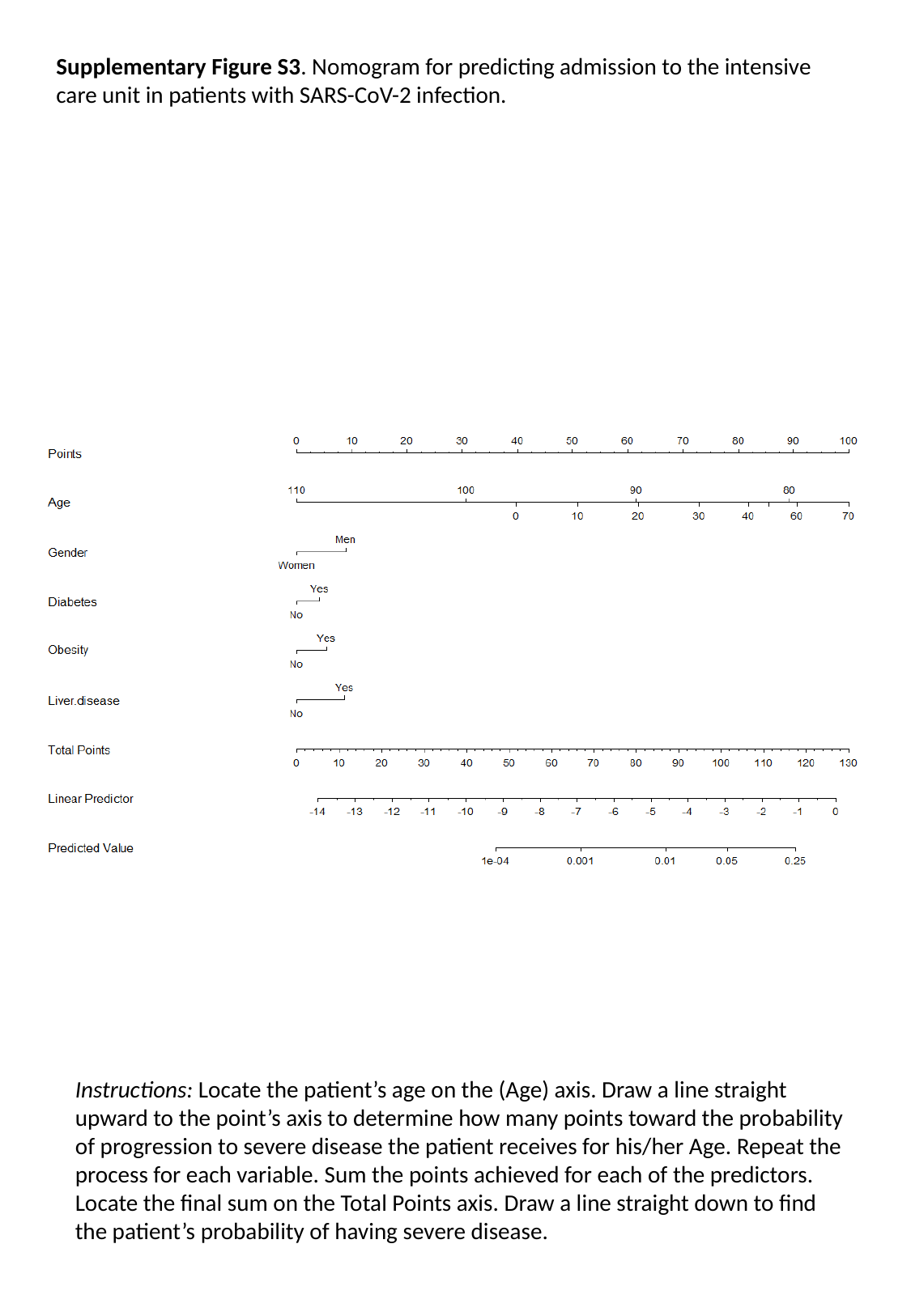

Supplementary Figure S3. Nomogram for predicting admission to the intensive care unit in patients with SARS-CoV-2 infection.
Instructions: Locate the patient’s age on the (Age) axis. Draw a line straight upward to the point’s axis to determine how many points toward the probability of progression to severe disease the patient receives for his/her Age. Repeat the process for each variable. Sum the points achieved for each of the predictors. Locate the final sum on the Total Points axis. Draw a line straight down to find the patient’s probability of having severe disease.

## Slide 4
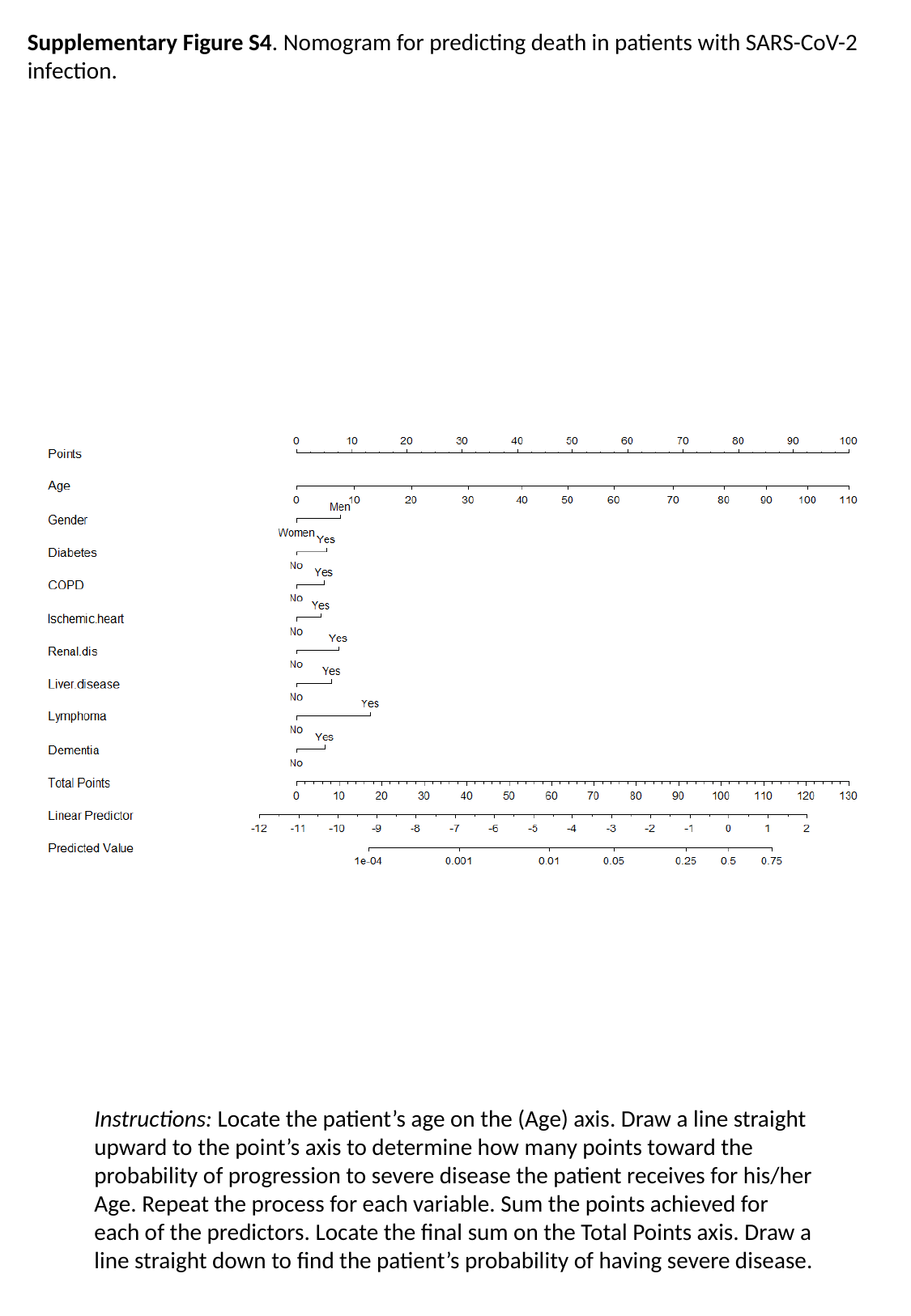

Supplementary Figure S4. Nomogram for predicting death in patients with SARS-CoV-2 infection.
Instructions: Locate the patient’s age on the (Age) axis. Draw a line straight upward to the point’s axis to determine how many points toward the probability of progression to severe disease the patient receives for his/her Age. Repeat the process for each variable. Sum the points achieved for each of the predictors. Locate the final sum on the Total Points axis. Draw a line straight down to find the patient’s probability of having severe disease.
